# From First Breathless Episode to Final Diagnosis and Treatment: A Case Report on Thoracic Endometriosis Syndrome

**DOI:** 10.3390/jcm14176240

**Published:** 2025-09-04

**Authors:** Katarzyna Pietrzak, Anna Weronika Szablewska, Bartosz Pryba, Aleksandra Gaworska-Krzemińska

**Affiliations:** 1 Independent Monoprofile Medical Simulation Laboratory, Institute of Nursing and Midwifery, Medical University of Gdańsk, Sklodowskiej-Curie 3A, 80-210 Gdańsk, Poland; bartosz.pryba@gumed.edu.pl; 2 Department of Obstetric and Gynaecological Nursing, Institute of Nursing and Midwifery, Medical University of Gdańsk, Sklodowskiej-Curie 3A, 80-210 Gdańsk, Poland; anna.szablewska@gumed.edu.pl; 3 Division on Nursing Management, Institute of Nursing and Midwifery, Medical University of Gdańsk, Sklodowskiej-Curie 3A, 80-210 Gdańsk, Poland; aleksandra.gaworska-krzeminska@gumed.edu.pl

**Keywords:** endometriosis, thoracic endometriosis syndrome (TES), cyclic chest pain, woman’s health, delayed diagnosis

## Abstract

**Background:** Endometriosis is a chronic disease defined by the presence of endometrial-like tissue outside the uterine cavity. While typically confined to the pelvis, extrapelvic manifestations—including thoracic endometriosis—can occur. Although rare, thoracic endometriosis is the most common extragenital form. In clinical practice, this presentation is often described as thoracic endometriosis syndrome (TES), a constellation of cyclic thoracic symptoms temporally associated with menstruation but not always histologically confirmed. Its atypical symptoms and limited clinical awareness frequently lead to delayed diagnosis, mismanagement and increased patient burden. **Methods:** In accordance with the CARE guidelines, we present a case report of a female patient with thoracic endometriosis syndrome, emphasizing the prolonged interval between symptom onset and final diagnosis. **Case Report:** We describe a 42-year-old woman with a longstanding history of dysmenorrhea and menorrhagia, who developed cyclical chest pain and dyspnea in 2019. Despite multiple thoracoscopic procedures, her symptoms persisted and were repeatedly misattributed to anxiety or infection. Thoracic endometriosis syndrome (TES) was suspected in 2022, and although histopathological confirmation was lacking, intraoperative visualization revealed diaphragmatic fenestrations. In 2025, a second laparoscopic intervention targeting the abdominal surface of the diaphragm resulted in significant symptom relief. The patient is currently continuing hormonal therapy with Dienogest and has reported a marked improvement in quality of life. Nevertheless, the protracted diagnostic and therapeutic process—marked by chronic pain and repeated hospitalizations—had a profound psychosocial impact, culminating in a suicide attempt. **Conclusions:** This case illustrates the substantial burden associated with the delayed recognition of thoracic endometriosis syndrome and the consequences of fragmented care. The patient’s experience underscores the urgent need for coordinated, multidisciplinary management and psychological support, particularly for patients with extrapelvic manifestations. Early multidisciplinary evaluation, with readiness to consider surgical intervention alongside individualized hormonal therapy, may support improved outcomes, provided they are reinforced by increased clinical awareness and systemic improvement in diagnostic pathways.

## 1. Introduction

Endometriosis is a systemic, inflammatory, estrogen-dependent condition characterized by endometrial stroma and gland-like lesions outside of the uterus. It affects approximately 10% of women of reproductive age and is associated with chronic pain, infertility and significant reductions in quality of life [1,2]. The condition also imposes a considerable economic burden due to healthcare costs and productivity loss. The extra-abdominal and pelvic manifestations of endometriosis have been described and account for about 12% of endometriosis cases [3]. Among the extragenital sites, the thorax is the most frequent location of endometriotic lesions. Thoracic endometriosis was described for the first time by Schwarz in 1938 [1]. The condition is diagnosed when endometrial tissue is identified in the lung parenchyma, pleura and diaphragm. The term thoracic endometriosis syndrome (TES) refers to clinical manifestations that occur in synchrony with menstruation, even in the absence of histological confirmation. In studies, it is indicated that up to 80% of individuals diagnosed with TES also present with coexisting pelvic endometriosis [1,2,3]. Pelvic involvement is typically diagnosed between the ages of 25 and 35, and it is believed to precede the onset of thoracic symptoms by approximately five to seven years. Catamenial pneumothorax is the most common manifestation (73%) of TES and is a rare, spontaneous and recurrent form of pneumothorax observed in women of reproductive age. It typically occurs between 24 h before menstruation and up to 72 h after its onset, although some sources extend this window to as long as seven days before or after menstrual bleeding [2,3]. While often associated with thoracic endometriosis, its occurrence may or may not coincide with a histological confirmation of the condition. The other manifestations include catamenial hemothorax (13%), catamenial hemoptysis (10%), shoulder pain, and, rarely, pulmonary nodules (4%) [1].

The diagnosis of thoracic endometriosis syndrome remains challenging due to its non-specific symptoms and variable presentation. Clinical suspicion is primarily based on patient history, particularly the presence of cyclical thoracic symptoms in reproductive-age women, often with coexisting pelvic endometriosis [4]. Imaging modalities such as chest X-ray, computed tomography (CT) or magnetic resonance imaging (MRI) can aid in ruling out other intrathoracic pathologies; however, they lack specificity for endometriotic lesions [5]. Definitive diagnosis relies on surgical exploration via video-assisted thoracoscopic surgery (VATS), with histopathological or immunohistochemical confirmation [2,4]. In many cases, endometrial stroma alone is identified, necessitating the use of markers such as the ER (Estrogen Receptor), the PR (Progesterone Receptor) and CD10 (Cluster of Differentiation 10) to support diagnosis. Emerging biomarkers, including CA-125 and those circulating endometrial cells, show promise but require further validation [6,7]. A multidisciplinary approach involving experienced pathologists and gynecologists is essential to ensure accurate identification and effective management. Women with atypical symptoms—such as cyclical chest pain or pneumothorax—may experience years of medical dismissal or incorrect treatment. Patients with endometriosis experience not only severe physical symptoms but also significant psychological distress [8]. Feelings of exclusion, anxiety tied to each menstrual cycle, depressive symptoms and relational difficulties are frequently reported by affected individuals.

Management of thoracic endometriosis is most often surgical and involves the resection of endometrial lesions from the pleura and/or diaphragm, typically via video-assisted thoracoscopic surgery. In patients who are candidates for VATS, simultaneous video-laparoscopic surgery may be recommended. As demonstrated by Nezhat et al. (2014) [6] in their retrospective observational study of 25 patients, it was reported that 76% of patients with TES had both pleural and abdominal diaphragm involvement. Pharmacological management of thoracic endometriosis syndrome prioritizes medical hormonal therapy when feasible. Therapeutic options include gonadotropin-releasing hormone (GnRH) analogs and antagonists, oral contraceptives, progestins and aromatase inhibitors [2,9].

Despite increasing interest, thoracic endometriosis remains underdiagnosed, and there is a clear knowledge gap in its early identification, especially in emergency and primary care settings. Women often report being dismissed or misdiagnosed for years, resulting in delayed treatment and psychological distress [6,10]. More attention is needed to recognize non-gynecological manifestations of endometriosis. Documenting such cases can help us develop better diagnostic guidelines and reduce the stigma faced by women with unexplained cyclical symptoms, ultimately improving outcomes through earlier, multidisciplinary care. Given the complex nature of this condition, patients must also be provided with psychological support and reassurance. All medical interventions should be implemented within a patient-centered, multidisciplinary care model to ensure the highest standard of care. Finally, nursing advocacy and health education—both among healthcare professionals and the general public—play a critical role in improving outcomes by reducing diagnostic delays and enhancing quality of life among women with thoracic endometriosis. This case report aims to present the clinical course, diagnostic challenges and multidisciplinary management of a female patient with a catamenial pneumothorax as a manifestation of thoracic endometriosis syndrome.

## 2. Case Report

In the report, the complexity of delayed diagnosis is underscored, as well as the impact of recurrent symptoms on the patient’s physical and psychological well-being. The need for increased clinical awareness and coordinated care across specialties is also highlighted. Written informed consent was obtained from the patient to publish this case report. The study was approved by the institutional bioethics committee (Approval No. KB/517/2023). The case is structured following CARE guidelines [11].

### 2.1. Patient Information

The patient is a 42-year-old woman with higher education, employed as a school teacher, married, and a mother of two children. The patient had no history of contraceptive use or assisted reproductive technologies. Her first pregnancy (2007) was delivered via cesarean section due to signs of fetal distress. The second pregnancy resulted in an uncomplicated vaginal delivery (2012). The patient had no history of chronic medical conditions. Her family history was notable for malignancies on the maternal side, including cervical as well as breast cancer. She is an only child. Since menarche in 1996, the patient has suffered from severe dysmenorrhea, menorrhagia and prolonged pelvic pain with bleeding episodes lasting up to seven days. In 2019, she developed recurrent chest pain and dyspnea. The patient’s basic clinical information is summarized in Table 1.

**Figure 1:**
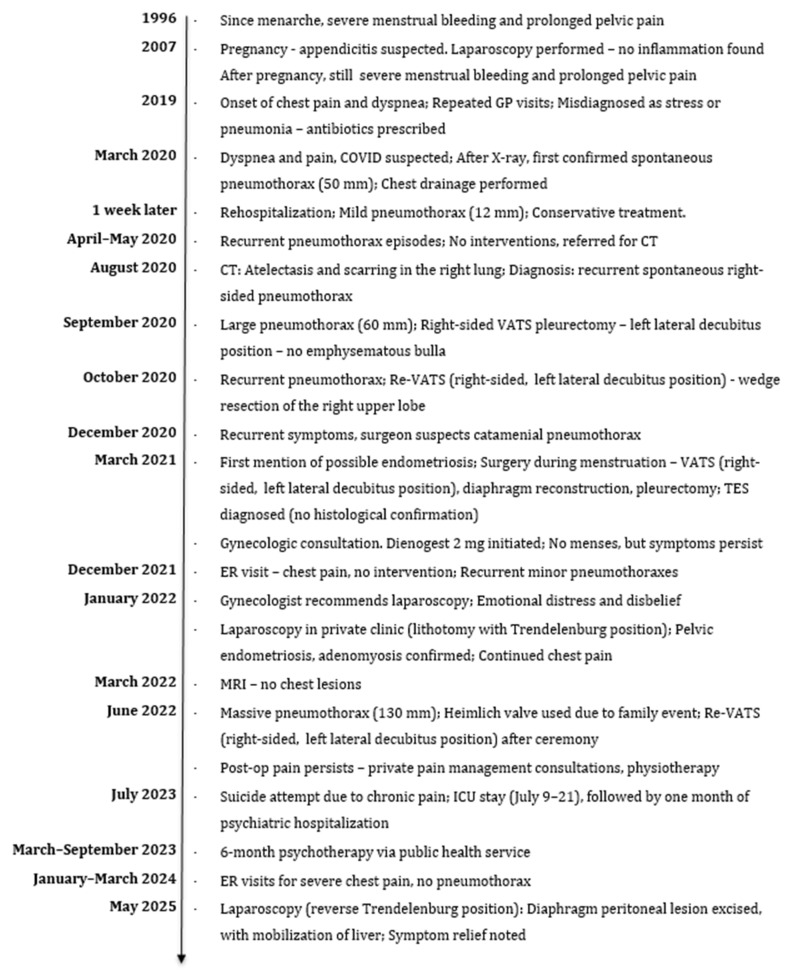
Patient’s medical history timeline.

The patient experienced significant emotional distress, frustration with the healthcare system, relationship difficulties and, eventually, a suicide attempt related to chronic pain in July 2023. In 2007, during pregnancy, the patient underwent a laparoscopy due to suspected appendicitis, which revealed no signs of inflammation. Following pregnancy, her symptoms of severe menstrual bleeding and pelvic pain persisted. Over the subsequent years, she required multiple interventions due to recurrent pneumothorax and, later, endometriosis (Figure 1).

### 2.2. Clinical Findings

**Figure 2:**
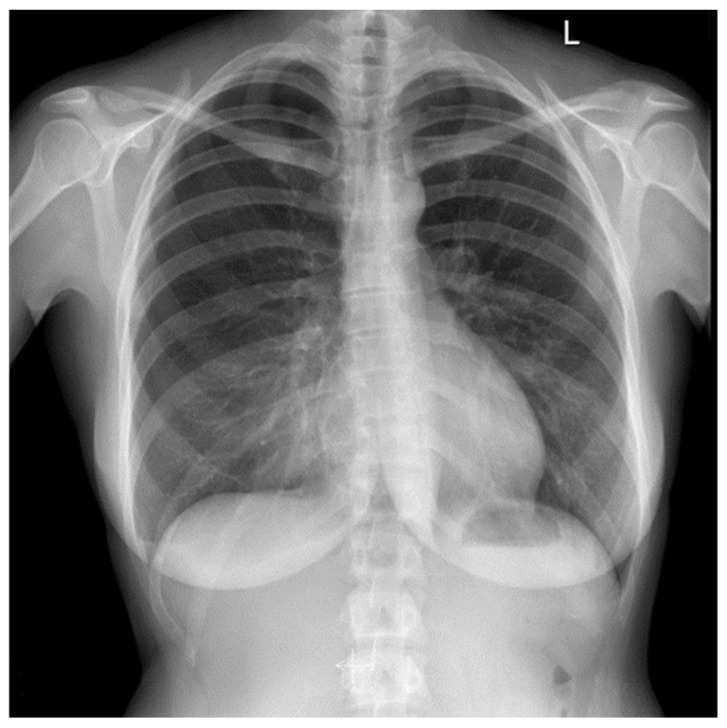
Right lung pneumothorax in September 2020 *(L—left side marker)*.

**Figure 3:**
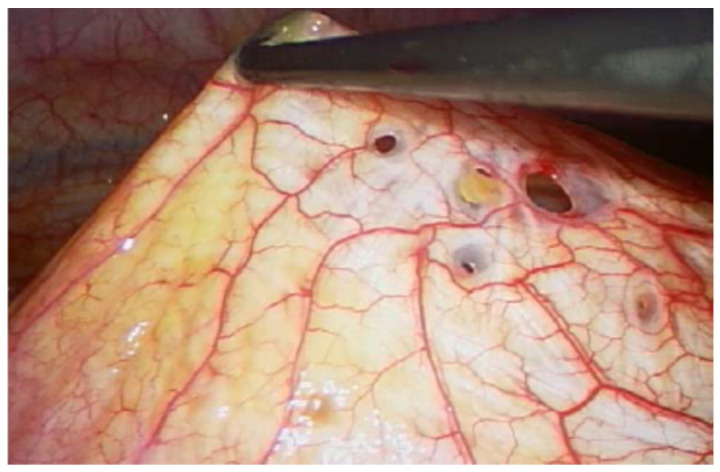
Thoracoscopic view of diaphragmatic fenestrations.

**Figure 4:**
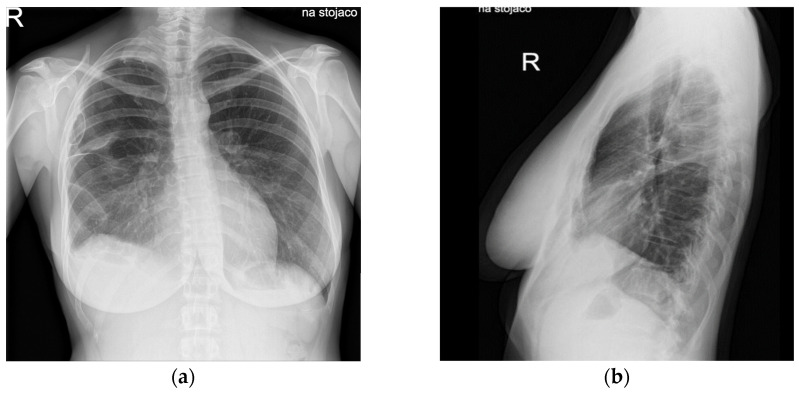
Chest X-ray showing pleural adhesions, fibrotic strands and increased subpleural markings in the right lung in August 2022: (**a**) PA projection; (**b**) right-lateral projection *(R—right side marker)*.

On physical examination, surgical scars were observed on the abdominal and thoracic skin, consistent with prior operative interventions. Auscultation of the chest revealed decreased vesicular breathing sounds. No additional detailed data from the physical examination at presentation were available. Initial findings included chest pain and dyspnea; imaging revealed a recurrent right-sided spontaneous pneumothorax (Figure 2). Diaphragmatic defects were identified during VATS in March 2021 (Figure 3). Pelvic endometriosis and stage 3 adenomyosis were later confirmed by laparoscopy in January 2022 in the uterosacral ligament and the rectovaginal septum. Pleural adhesions, fibrotic changes and increased subpleural markings were noted in the mid-to-lower right lung field in August 2022 (Figure 4).

### 2.3. Timeline

Historical and current information is summarized below. See Figure 1 for a visual timeline. On chest radiography, the pneumothorax was reported as measuring in millimeters as documented by the radiologist; however, the specific measurement method was not specified in the medical records.

### 2.4. Diagnostic Assessment and Therapeutic Intervention

Diagnostic assessment included repeated imaging—such as chest X-rays, computed tomography (CT) and magnetic resonance imaging (MRI)—as well as multiple laparoscopic and video-assisted thoracoscopic (VATS) procedures. From the onset of symptoms, the diagnostic process was challenging: the patient’s complaints were repeatedly attributed either to psychological causes, such as anxiety, or to suspected infections, including pneumonia. These initial misattributions led to a significant delay in the recognition of thoracic endometriosis syndrome (TES). In the early stages of her illness, the patient was also evaluated for suspected appendicitis. Differential diagnoses considered over the years included anxiety, pneumonia, appendicitis and non-specific pain. In 2022, the surgical procedure was deliberately scheduled during menstruation in an attempt to increase the likelihood of identifying ectopic endometrial tissue. Standard thoracoscopic inspection revealed multiple diaphragmatic fenestrations, which were repaired using size 5 braided, synthetic, absorbable sutures. Inspection under saline with positive pressure was performed, without revealing microperforations. Additional areas, including the costophrenic recesses, diaphragm, mediastinal pleura and pulmonary pleura, were irritated with gauze soaked in 10% NaCl to promote pleurodesis. Pleural and diaphragmatic specimens were submitted for histopathological evaluation. No histological confirmation was achieved. Magnetic resonance imaging (MRI) was performed once in three planes during menstruation, before and after intravenous contrast administration, including Diffusion Weighted Imaging (DWI)/Apparent Diffusion Coefficient (ADC) sequences; no fat-suppressed T1-weighted images were obtained, and the method also failed to reveal any abnormalities within the thoracic cavity. Intraoperatively, diaphragmatic defects were visualized and subsequently repaired (Figure 4). Despite prior surgical intervention involving diaphragmatic reconstruction, pleurectomy and chemical pleurodesis, the patient developed a massive pneumothorax in June 2022, necessitating repeat surgical management. The patient was informed by the thoracic surgeon about the risk of pneumothorax recurrence following the surgical intervention.

Therapeutic management was multidisciplinary. Pharmacologically, Dienogest 2 mg daily was initiated after March 2021 and continued postoperatively in response to persistent symptoms. Surgical interventions included chest drainage for pneumothorax, VATS pleurectomy, wedge resection of the right upper lobe, diaphragm reconstruction and laparoscopic procedures targeting both endometriosis involving the pelvic organs/peritoneum and the diaphragm. During laparoscopy in May 2025, mobilization of the liver was undertaken to fully expose the right hemidiaphragm. The altered peritoneum overlying the right diaphragmatic dome was excised. The left ovarian vein was clipped; however, the indication was not explicitly documented. This finding is reported for completeness, as the potential role of pelvic venous congestion in TES remains unclear. Precise values of insufflation pressures were not documented. Excision specimens from the peritoneum were sent for standard histopathological evaluation. No histological confirmation of endometriosis was achieved. The surgical approach was adjusted over time based on site and recurrence of disease manifestations.

In addition to medical and surgical treatments, the patient underwent consultations for pain management and physiotherapy. Prescribed analgesic therapy included Oxycodone + Naloxone 10 mg + 5 mg twice daily and Pregabalin 75 mg twice daily. After the suicide attempt—related to chronic pain—the patient was referred for supportive psychotherapy, including both cognitive-behavioral and psychodynamic approaches. Throughout the clinical course, management strategies were individualized and adapted according to symptom recurrence and disease progression, with the ongoing use of Dienogest remaining central to pharmacological therapy.

### 2.5. Follow-Up and Outcomes

After the laparoscopic surgery performed in May 2025, the patient reported a significant reduction in symptoms, with marked improvement in both chest pain and dyspnea. These complaints, which had previously recurred cyclically and often required emergency interventions, became much less frequent and less intense. Since the operation, no further acute hospital visits were necessary. The patient was able to return to full-time employment as a school teacher and described a notable improvement in her overall quality of life and daily functioning. She continues to experience only occasional, mild chest discomfort, which does not interfere with her regular activities. Both the patient and her clinical team have noted and documented this substantial clinical improvement. The patient maintains ongoing hormonal therapy with Dienogest and attends regular gynecological and thoracic follow-up appointments. To date, she has not experienced any major adverse effects or other unanticipated events related to her therapy or subsequent recovery. It is important to note that information regarding follow-up imaging or laboratory studies after May 2025 is currently lacking. The only major adverse event reported during her treatment course was a suicide attempt in July 2023, related to chronic pain and psychological distress. Since her most recent surgery, the patient reported a marked reduction in thoracic symptom burden. In the 12 months preceding the procedure, she experienced severe recurrent chest pain, typically rated as 8–10 on the numerical rating scale (NRS), and required multiple emergency visits for acute exacerbations. She also missed approximately ten work days due to thoracic pain in this period. Since the surgery, and up to the last follow-up in August 2025, she has remained free of chest pain in daily life and has not required emergency care or work leave. Pain associated with menstruation could not be reassessed, as the patient is currently amenorrheic while receiving hormonal therapy (Dienogest). At present, she has achieved a three-month recurrence-free interval.

## 3. Discussion

In the presented case, the pivotal importance is underscored of timely diagnosis and intervention in thoracic endometriosis, as well as the necessity of coordinated, multidisciplinary management to optimize patient outcomes. In this patient, the combination of delayed diagnosis, numerous healthcare visits, self-financed investigations, persistent pain and anxiety related to breathlessness resulted in significant mental health consequences. Addressing these challenges is crucial for improving both the physical and psychological burden of endometriosis.

Extrapelvic endometriosis most frequently affects the thoracic cavity, followed by sites such as the bowel, omentum, urinary tract and central nervous system [6]. A 2019 systematic review by Andres et al. demonstrated that the thorax accounts for the majority of reported extrapelvic endometriosis cases, with approximately 628 out of 920 cases identified in this region [12,13]. Thoracic endometriosis is typically diagnosed in women around the age of 35 and has been reported in up to 30% of women undergoing surgery for spontaneous pneumothorax [14]. However, it remains unclear whether this reflects a truly late onset of the disease or rather a prolonged diagnostic delay [1]. The striking right-sided predominance of thoracic endometriosis aligns with the leading mechanistic hypotheses described in the literature [15]. Proposed explanations include peritoneal fluid dynamics directing endometrial cells preferentially along the right paracolic gutter, the presence of congenital or acquired fenestrations in the tendinous portion of the right hemidiaphragm that facilitate translocation, and the so-called ‘hepatic barrier effect’, whereby the liver impedes the spread of endometrial cells to the left hemidiaphragm. Together, these mechanisms provide a plausible pathophysiologic rationale for the lateralization observed in this case.

Accurate diagnosis of thoracic endometriosis often requires several recurrent clinical episodes before confirmation. In the literature, an average interval is reported of approximately 19 months between the initial episode of pneumothorax and a definitive diagnosis of thoracic endometriosis [16]. A retrospective review of medical records from an endometriosis surgery center identified thoracic endometriosis syndrome (TES) in 22 patients (2.6%) who underwent surgical procedures between May 2019 and June 2023. In this cohort, the average age at onset of thoracic symptoms was 33 years, typically occurring about 13 years after the initial pelvic symptoms. Patients consulted an average of 6.7 providers and underwent 2.5 procedures before receiving the correct diagnosis, with a mean diagnostic delay of 14 years [17].

Important elements in the patient’s medical history may include the onset of symptoms in temporal association with menstruation, a predominance of right-sided thoracic involvement, reproductive age, recurrent clinical episodes and a prior history of infertility [13]. Looking ahead, mobile applications and digital platforms may become effective tools for monitoring symptoms and supporting the diagnostic process in endometriosis and related conditions (e.g., Endometriosis Risk Advisor–Nezhat Endometriosis Advisor, version 1.7.2 (Inframatic LLC, Mechanicsburg, PA, USA), potentially facilitating earlier recognition and improved patient outcomes [18].

In the diagnostic evaluation of thoracic endometriosis syndrome (TES), magnetic resonance imaging (MRI) of the chest and abdomen is currently regarded as the most appropriate preoperative imaging modality [16]. When using fat-suppressed T1-weighted sequences, MRI demonstrates a reported sensitivity of 78–83% for detecting diaphragmatic endometriosis, to markedly lower values (around 30–50%) when imaging is obtained outside the symptomatic timeframe or without dedicated protocols. The presence of the so-called “air-filled bubble” sign may indicate small perforations in the diaphragm. These figures highlight that accuracy is both operator- and timing-dependent. In our patient, MRI obtained in the intermenstrual phase was interpreted as normal, yet subsequent thoracoscopic inspection revealed multiple diaphragmatic fenestrations consistent with thoracic endometriosis syndrome [19]. Performing MRI, both during menstruation and mid-cycle, can help identify subtle changes, as previously observed findings may resolve between phases, thereby reinforcing clinical suspicion of thoracic endometriosis syndrome [20]. However, in our patient, MRI performed during menstruation did not reveal any abnormalities suggestive of diaphragmatic endometriosis, highlighting the diagnostic challenges and potential limitations of imaging—even when conducted at an optimal timing.

Peripheral blood-based biomarkers have also attracted interest in the diagnostic evaluation of thoracic endometriosis syndrome (TES). Recent evidence suggests that circulating endometrial cells could serve as a promising non-invasive marker in this context. In a recent study, Kiss et al. identified such cells in the peripheral blood of patients with suspected catamenial pneumothorax. These cells exhibit hormone-dependent cytomorphological changes and distinct gene expression profiles, potentially enabling earlier detection of thoracic involvement and helping to reduce diagnostic delays in future clinical practice [21].

Several factors may influence therapeutic decision-making, including medication cost, side-effect profiles, accessibility and individualized treatment objectives. Consideration of the patient’s reproductive plans, particularly the desire for future childbearing, is also essential in guiding management [22]. The European Society of Human Reproduction and Embryology (ESHRE) guidelines recognize hormonal therapy—such as combined oral contraceptives, progestins and GnRH analogs—as viable first-line options for extrapelvic endometriosis, including TES, particularly when surgery is contraindicated or undesired [23]. Subsequent management is guided by the degree of clinical suspicion. According to a recent meta-analysis by Ciriaco et al. [16], in cases with high suspicion, simultaneous video-assisted thoracoscopic surgery (VATS) and laparoscopy are preferred to allow for comprehensive assessment of both thoracic and pelvic compartments. In patients with low or moderate suspicion, VATS is initially performed, followed by staged laparoscopy, if indicated. In both approaches, the goal is complete excision of all visible endometriotic lesions, followed by histopathological confirmation. In the diagnosis of TES, Mecha et al. [4] demonstrated that stromal endometriosis was detected in 52% of cases via immunohistochemistry (ER, PR, CD10), compared to just 10% with conventional histology, suggesting a much higher diagnostic yield using IHC—even CD10 alone. This supports the value of routine IHC when tissue is available in suspected TES. In our case, however, only standard histopathological evaluation was performed, without immunohistochemical analysis, which may have limited the diagnostic sensitivity. Postoperatively, patients should undergo gynecologic evaluation for the initiation of hormonal suppression therapy, and long-term follow-up should be coordinated jointly by thoracic surgery and gynecology specialists [16].

Accurate diagnosis and effective management of diaphragmatic endometriosis require thorough and unobstructed visualization of the entire diaphragm. In a recent review by Andres et al., among 628 patients with thoracic endometriosis syndrome (TES) evaluated during VATS, endometrial lesions were most frequently identified on the diaphragm (78.82%), followed by the pleura (14.33%), lung parenchyma (4.46%), and all three sites concurrently in 1.11% of cases [24,25]. In the case of patients eligible for video-assisted thoracoscopic surgery (VATS), a combined approach with simultaneous laparoscopy may be advisable. In a retrospective observational study from 2014 involving 25 patients, it was found that 76% of those diagnosed with TES exhibited involvement of both the pleural and abdominal surfaces of the diaphragm [6,26]. In our patient, VATS was performed four times without achieving sustained symptom relief. In 2025, a second laparoscopic procedure was undertaken, during which the diaphragmatic surface was addressed from the abdominal side and the liver was mobilized. In this case, a staged strategy was implemented given acute thoracic indications and non-diagnostic preoperative imaging; once persistent cyclic symptoms raised concerns for residual abdominal-side diaphragmatic disease, a targeted gynecologic laparoscopy was performed. This sequencing may have influenced recurrence dynamics and is discussed in the context of multidisciplinary care. In this patient, it ultimately led to a marked alleviation of symptoms.

Since 2007, the British Society for Gynaecological Endoscopy (BSGE) has designated specialist centers to provide standardized care for patients with severe endometriosis, based on strict clinical and procedural criteria [27]. Despite this structured approach, disparities in access and consistency of care persist, as reported by patients with thoracic endometriosis—some of whom have described conflicting guidance received even within BSGE-accredited centers. Although awareness of endometriosis as a significant public health issue is growing, access to multidisciplinary care remains uneven globally. In many countries, comprehensive management—including timely diagnosis and coordinated treatment involving gynecologists, pain specialists, surgeons, physiotherapists and dietitians—continues to depend on local resources or individual institutions rather than national frameworks [28]. As a result, patients frequently face long delays before reaching specialized centers, which may further complicate their clinical outcomes [17,27]. Efforts to develop structured care pathways and specialized clinics are underway in several regions, but the effectiveness and accessibility of these initiatives continue to be evaluated. In the present case, the patient did not have access to a comprehensive, coordinated care model in which each specialist could contribute within their area of expertise to provide truly integrated management. Instead, she was forced to independently seek care from multiple specialized centers, often incurring substantial out-of-pocket expenses. This fragmented approach significantly prolonged the interval between the onset of symptoms and the final diagnosis, while also having profound, negative impact on her quality of life.

Systemic treatment of endometriosis demonstrates notable variability across countries, reflecting differences in clinical guidelines and healthcare access. In the review by Kalaitzopoulos et al. (2021), six national (France, Germany, Canada, United Kingdom, United States) and two international guidelines were analyzed, revealing no uniform consensus regarding the optimal approach to systemic treatment of extrapelvic endometriosis [29]. Current evidence-based strategies for the diagnosis and management of thoracic endometriosis syndrome (TES) remain underdeveloped, largely due to the low prevalence of the condition and the limited quality of existing research. Advancing knowledge in this area would benefit from the establishment of a dedicated registry, supported by multidisciplinary collaboration among teams of gynecologists, general as well as thoracic surgeons [7].

Endometriosis imposes a considerable social and psychological burden on affected women, influencing multiple aspects of their lives. Diagnostic delays are associated with reduced health-related quality of life [13]. In our patient, the prolonged diagnostic and therapeutic process—marked by chronic severe pain and multiple hospitalizations—had profound emotional and psychological impact. The lack of symptom relief and uncertainty regarding her condition led to feelings of helplessness and despair, ultimately resulting in a suicide attempt. In existing research, it is indicated that endometriosis is associated with a wide range of psychological consequences, including emotional distress, social isolation, feelings of hopelessness and low self-worth, as well as symptoms of depression and suicidal ideation. Considering the potential influence of endometriosis on mental health, social functioning and financial toxicity due to work absence, further investigation in this area would be highly valuable [30].

This case emphasizes the need for heightened clinical suspicion of thoracic endometriosis syndrome in patients with unexplained cyclical thoracic symptoms. It demonstrates the practical value of early, coordinated thoracic and gynecologic assessment to optimize diagnosis, reduce recurrence and guide individualized treatment strategies.

Future research should aim to standardize imaging protocols for suspected TES, including optimal MRI sequences and timing across the menstrual cycle. Moreover, prospective registries or multicenter collaborations are needed to better define clinical predictors, surgical outcomes and long-term recurrence rates. The absence of randomized trials highlights both the difficulty and necessity of structured research in this rare condition.

## 4. Conclusions

Thoracic endometriosis syndrome should be considered in all women of reproductive age presenting with unexplained pneumothorax, especially when symptoms align with the menstrual cycle. Early surgical intervention through VATS and laparoscopy may not only confirm the diagnosis, but also greatly enhance the patient’s quality of life. This case emphasizes the importance of multidisciplinary collaboration in addressing both the physical and emotional needs of patients with TES. The urgent need for comprehensive psychosocial support in the management of patients with endometriosis is highlighted. This is particularly significant in cases with complex and extrapelvic presentations. Despite recurrent, cyclical respiratory symptoms, the patient’s complaints were often dismissed or misattributed, leading to years of untreated illness and psychological distress.

While our patient’s trajectory highlights the potential benefit of operative intervention, it is important to acknowledge the low quality of evidence in thoracic endometriosis, the absence of randomized trials, and the potential for selection as well as publication bias in the surgical literature. On this basis, we recommend early multidisciplinary evaluation with readiness for combined operative management when clinical suspicion is high, rather than positioning surgery as uniformly superior to medical therapy.

## Figures and Tables

**Table 1:** Basic clinical data of the patient.

Characteristic	Value
Age at diagnosis of endometriosis (years)	37
Body Mass Index (BMI) at diagnosis * (kg/m^2^)	23 kg/m^2^
Age at first menstruation (years)	14
Age at first pregnancy (years)	24
Number of pregnancies	2
Number of live births	2
Number of miscarriages	0
Characteristics of menstrual cycle	Irregular cycles, 30/31 days, heavy, long (~7 days), very painful, sometimes bedridden since school age.
Previous gynecological surgeries	Curettage before first pregnancy in age of 23
Comorbidities	Depression
Family history of endometriosis	None
Family history of other chronic diseases	Maternal grandmother—hypertension; Mother—malignant cervical and breast cancer.
Smoking	No
Alcohol consumption	Yes—occasional
Allergies	Inhalant—grass pollen, dust
Medications at the time of endometriosis diagnosis	None

* BMI (Body Mass Index) calculated as weight (kg)/height^2^ (m^2^).

## Data Availability

The data presented in this study are available within the article. Further details are not publicly available due to concerns regarding patient privacy.

## Abbreviations

The following abbreviations are used in this manuscript:

TES	Thoracic Endometriosis Syndrome
VATS	Video Assisted Thoracoscopy
GnRH	Gonadotropin-releasing Hormone
BSGE	British Society for Gynecological Endoscopy
MRI	Magnetic Resonance Imaging
ER	Estrogen Receptor
PR	Progesterone Receptor
DWI	Diffusion Weighted Imaging
ADC	Apparent Diffusion Coefficient
